# The Delta-Subunit Selective GABA_*A*_ Receptor Modulator, DS2, Improves Stroke Recovery via an Anti-inflammatory Mechanism

**DOI:** 10.3389/fnins.2019.01133

**Published:** 2019-10-29

**Authors:** Silke Neumann, Lily Boothman-Burrell, Emma K. Gowing, Thomas A. Jacobsen, Philip K. Ahring, Sarah L. Young, Karin Sandager-Nielsen, Andrew N. Clarkson

**Affiliations:** ^1^Department of Pathology, Dunedin School of Medicine, University of Otago, Dunedin, New Zealand; ^2^Department of Anatomy, Brain Health Research Centre, Brain Research New Zealand, University of Otago, Dunedin, New Zealand; ^3^Saniona A/S, Copenhagen, Denmark; ^4^School of Pharmacy, University of Sydney, Sydney, NSW, Australia

**Keywords:** stroke, inflammation, GABA, immune response, neuroinflammation, functional recovery, neuroimmunology

## Abstract

Inflammatory processes are known to contribute to tissue damage in the central nervous system (CNS) across a broad range of neurological conditions, including stroke. Gamma amino butyric acid (GABA), the main inhibitory neurotransmitter in the CNS, has been implicated in modulating peripheral immune responses by acting on GABA_*A*_ receptors on antigen-presenting cells and lymphocytes. Here, we investigated the effects and mechanism of action of the delta-selective compound, DS2, to improve stroke recovery and modulate inflammation. We report a decrease in nuclear factor (NF)-κB activation in innate immune cells over a concentration range *in vitro*. Following a photochemically induced motor cortex stroke, treatment with DS2 at 0.1 mg/kg from 1 h post-stroke significantly decreased circulating tumor necrosis factor (TNF)-α, interleukin (IL)-17, and IL-6 levels, reduced infarct size and improved motor function in mice. Free brain concentrations of DS2 were found to be lower than needed for robust modulation of central GABA_*A*_ receptors and were not affected by the presence and absence of elacridar, an inhibitor of both P-glycoprotein and breast cancer resistance protein (BCRP). Finally, as DS2 appears to dampen peripheral immune activation and only shows limited brain exposure, we assessed the role of DS2 to promote functional recovery after stroke when administered from 3-days after the stroke. Treatment with DS2 from 3-days post-stroke improved motor function on the grid-walking, but not on the cylinder task. These data highlight the need to further develop subunit-selective compounds to better understand change in GABA receptor signaling pathways both centrally and peripherally. Importantly, we show that GABA compounds such as DS2 that only shows limited brain exposure can still afford significant protection and promote functional recovery most likely via modulation of peripheral immune cells and could be given as an adjunct treatment.

## Introduction

Stroke is the leading cause of lasting disability, with patients experiencing varied levels of functional recovery, and with more than 50% of survivors being discharged into care (Dobkin, 2008; Go et al., 2014). Changes in neuronal excitability, loss of gamma amino butyric acid (GABA) inhibition, enhanced glutamatergic signaling, and changes in neuronal connections and plasticity all contribute to impairment after stroke (Wittenberg and Schaechter, 2009; Clarkson et al., 2010, 2011, 2015, 2019; Carmichael, 2012; Krakauer et al., 2012). In addition, it is well documented that the full expansion of the infarction and ongoing impairment in the weeks to months following a stroke is underpinned by inflammation and the infiltration of peripheral immune cells that cross the blood–brain-barrier (BBB) (Gelderblom et al., 2009, 2015; Doyle et al., 2015). To date, drug therapies have attempted to minimize the extent of cell death, however, all drug therapies that have been trialled have failed to translate into the clinic. Therefore, new drug therapies need to be developed in order to support the recovery of stroke patients.

Changes in inflammatory processes is a hallmark for many pathologies including obesity, diabetes and stroke. Although acute inflammation is beneficial for the repairing and healing process, chronic inflammation contributes to tissue damage. Immune cells play a critical role in contributing to brain damage initiated by ischemic stroke. As a consequence of stroke, immune cells migrate to the brain in response to danger signals (damage-associated molecular patterns, DAMPs), in an effort to repair the damage (Brait et al., 2010; Gelderblom et al., 2015). However, these cells can also promote further inflammation and damage. In addition, the injured brain has an immune-suppressive effect that promotes life-threating infections, which threaten the survival of stroke patients (Liesz et al., 2015).

Traditionally considered a disease refined to the brain, it is becoming increasingly clear that the immune system heavily impacts the pathology of stroke. Local microglia, endothelial cells, neurons, and astrocytes recognize danger signals released from dying cells, which in turn stimulate the production of pro-inflammatory cytokines that attract circulating immune cells to migrate to the central nervous system (Offner et al., 2006; Gelderblom et al., 2009). Together, local and infiltrating cells, contribute to further neural cell death by producing pro-inflammatory cytokines, reactive oxygen species and by activating matrix metalloproteinases (Amantea et al., 2015). In particular, microglia have been shown to be chronically activated even when the initial DAMPs have been cleared (Huh et al., 2003; McGeer et al., 2003). This results in prolonged neuroinflammation that is associated with delayed recovery in stroke patients (Liguz-Lecznar and Kossut, 2013), and impaired memory, sensory learning and plasticity (Greifzu et al., 2011; Doyle et al., 2015).

Gamma amino butyric acid, known for its role as inhibitory neurotransmitter in the CNS, has a similar inhibiting effect on immune cells thereby creating a link between the CNS and the peripheral inflammatory response (Reyes-Garcia et al., 2007). Both, innate and adaptive immune cells express functional GABA receptors and possess enzymes to synthesize and catabolize GABA (Wheeler et al., 2011; Fuks et al., 2012). This includes microglia, macrophages, dendritic cells and T-cells. GABA signals through GABA_*A*_ and GABA_*B*_ receptors, both of which are expressed on immune cells (Kuhn et al., 2004; Wheeler et al., 2011; Fuks et al., 2012). The composition of the five subunits that make up GABA_*A*_ receptors likely varies for the various immune cells, which in turn will account for differences in potency and efficacy of drug treatments targeting GABA receptors and GABA itself (Fuks et al., 2012). GABA is known to act on GABA_*A*_ receptors in both millimolar and nanomolar to micromolar concentrations depending on the location (synaptic versus extrasynaptic) and functional composition of the receptors (Mody, 2001; Semyanov et al., 2003; Glykys and Mody, 2007). Of importance, submicromolar GABA concentrations have not only been found around neurons in the brain, but have also been detected in blood and hormone-producing cells in the intestine (Petty et al., 1999; Braun et al., 2004; Wendt et al., 2004). In addition to being exposed to chronic low levels of GABA, these peripheral tissues and receptors are likely to also be modulated following treatment with various GABA modulators. With the development of subunit specific GABA modulators, we may be able to find and develop compounds that could selectively regulate the function of peripheral immune cells.

Extrasynaptic GABA_*A*_ receptors, which are located outside the synapse typically contain either the δ- or α5-subunit and are highly sensitive to low GABA concentrations (Mody, 2001). Recent evidence has shown that modulation of extrasynaptic GABA_*A*_ receptors plays an important role in minimizing the extent of damage when given early (within hours) to increase tonic GABA currents after a stroke. In addition, this modulation can also facilitate an improvement in motor function when treatment is initiated at a delay (days) to dampen tonic GABA currents after the initial insult (Clarkson et al., 2010, 2019). As little is known about the role of δ-containing GABA_*A*_ receptor after stroke, we were interested in testing the therapeutic effects of the δ-subunit-selective GABA_*A*_ receptor modulator DS2 (4-chloro-N-[2-(2-thienyl)imidazo[1,2-a]pyridin-3-yl]benzamide). DS2 positively modulates δ-containing GABA_*A*_ receptors (Wafford et al., 2009), however, DS2 has not been investigated in a clinical disease model. Therefore, we aimed to assess the potential of DS2 to improve stroke recovery and to modulate inflammatory responses in innate immune cells. Herein, we show that positive allosteric modulation of δ-containing GABA_*A*_ receptors with DS2 affords significant protection and improves motor function in a mouse model of stroke. Investigation into a potential mechanism of action revealed that DS2 reduces the activation of NF-κB in LPS-stimulated macrophages and reduces the expression of activation markers on bone marrow-derived dendritic cells (BMDCs). Interestingly, we show that DS2 only has limited brain exposure, indicating that DS2-mediated effects *in vivo* are most likely attributed to modulation of peripheral immune cells.

## Materials and Methods

### Materials

Lipopolysaccharides from *Escherichia coli* 055:B5 (LPS) were ordered from Sigma-Aldrich (St. Louis, MO, United States); Dulbecco’s Modified Eagle Medium (DMEM), 2-Mercapto-ethanol, Penicillin/Streptomycin, and Roswell Park Memorial Institute Medium (RPMI) were ordered from Life Technologies (Auckland, New Zealand); foetal calf serum (FCS) was purchased from Moregate Biotech (Hamilton, New Zealand), DS2 from Tocris Bioscience (Bristol, United Kingdom), Zeocin^TM^ from Invitrogen (Auckland, New Zealand) and Quantiblue^TM^ from InvivoGen (CA, United States). The LIVE/DEAD^®^ Fixable Near-IR Dead Cell Stain was purchased from Thermo Fisher Scientific (MA, United States); Granulocyte-macrophage colony-stimulating factor (GM-CSF) and flow antibodies MHCII FITC, CD80 PE, CD86 PE-Cy7, CD11c BV421, and CD40 APC came from BioLegend (Auckland, New Zealand). The reagents for real-time PCR (qPCR) were purchased from the following suppliers: RNeasy^®^ Plus Mini kit from Qiagen (Austin, TX, United States), DNA-free^TM^ kit from Life Technologies Corporation (Carlsbad, CA, United States), SensiFAST cDNA Synthesis Kit and SYBR Green kit from Bioline (London, United Kingdom) and primers ordered from Integrated DNA Technologies (Singapore).

### Cell Culture of RAWblue^TM^ Macrophage Cells

RAWblue^TM^ macrophages were cultured in DMEM supplemented with heat-inactivated FCS (10%) and 200 μg/mL Zeocin^TM^ (antibiotic selection) as previously described (Clarkson et al., 2019). For experiments, 5 × 10^4^ RAWblue macrophages were added to a 96-well plate (flat-bottom) in the absence of Zeocin, and left untreated for 16 h. Increasing concentrations of DS2 (10^–6^ to 10^–3^ M) were added for 30 min before LPS (2.5 ng/mL) was added. The supernatant was collected after 6 h and the levels of the secreted embryonic alkaline phosphatase (SEAP) determined using the Quantiblue^TM^ assay. For this, 80 μL of the Quantiblue solution and 30 μL of the cell culture supernatant were combined, left at 37°C for 3 h and the absorbance read at 620 nm. Three independent experiments were performed in triplicate each.

### Generation and Stimulation of Bone Marrow-Derived Dendritic Cells

Bone marrow-derived dendritic cells were generated from bone marrow cells obtained from C57Bl/6J mice as described previously (Scheicher et al., 1992). Briefly, bone marrow cells were isolated from the hind legs of mice and processed into single cell suspensions. Bone marrow cells were re-suspended in IMDM supplemented with 5% FCS, 20 ng/mL GM-CSF, 1% streptomycin/penicillin and 0.1% 2-mercapto ethanol, plated at a density of 5 × 10^5^ cells/mL and maintained at 37°C for 6 days. Half of the media was refreshed on days 3 and 5 of the culture. On day 6, BMDCs were seeded to a 96 well plate at 2 × 10^5^ cells/mL and either pre-incubated with DS2 (10^–8^ to 10^–4^ M) for 30 min or left untreated before stimulation with LPS (100 ng/mL). BMDCs were harvested and stained with antibodies against cell surface markers: CD11c BV421, MHCII FITC, CD80 PE, CD86 PE-Cy7, and CD40 APC after unspecific binding was blocked by using the CD16/32 antibody. Dead cells were excluded by gating on LIVE/DEAD^®^ Fixable Near-IR-negative cells. Fluorescence minus one controls for each fluorophore were used to distinguish positive from background staining.

### Quantitative Polymerase Chain Reaction (qPCR)

RAWblue macrophages were grown to approximately 80% confluency in 6-well plates and detached using a cell scraper. Cells were spun down to remove residual cDMEM and total RNA was extracted using the RNeasy^®^ Plus Mini kit as previously described (Clarkson et al., 2019). Total RNA was incubated with DNase using the DNA-free^TM^ kit before reverse transcription into cDNA was performed on a ThermoCycler using the SensiFAST cDNA Synthesis Kit (25°C for 10 min, 42°C for 15 min, and 85°C for 5 min). All kits were used according to the manufacturer’s instructions and the cDNA was stored at −20°C until further processing. qPCR samples were prepared by mixing the SYBR green master mix with RNAfree water and primers in a 96-well plate. All primers are listed in Table 1. qPCR was performed on a Roche LightCycler^®^ (30 s at 95°C, 40 cycles of 5 min at 95°C, 15 s at 60°C and 10 s at 72°C) and the LightCycler 480^®^ software was used to determine *Ct* values. A dissociation curve analysis was performed and the differences in *Ct* values was calculated using the following equation: 2^(Ctreference – CTtarget)^ (Schmittgen et al., 2000). *Ct* values were normalized against the house keeping gene SDHA. Dissociation curve analysis was performed for all samples (60 s at 95°C, 30 s at 55°C, and 30 s at 95°C).

**TABLE 1:** Primer sequences for GABA receptor subunits and transporters as determined by qPCR.

**Target gene**	**Primer sequences (5′–3′)**	**Amplicon length**
α1	F: CATGACAGTGCTCCGGCTAA	136
	R: GCCATCCTCTGTGATACGCA	
α2	F: TTCAAAGCCACTGGAGGAAAAC	107
	R: GCAGCAGAGACCATACATTGC	
α3	F: TCCTGCTGAGACCAAGACCT	361
	R: GGCTCAATCCAAGCAATGTT	
α4	F: GCCAAACCCTGGTAAAGGGA	99
	R: CGGGTCAGCGACTTCAAAAC	
α5	F: GCTGACCCATCCTCCAAACA	71
	R: TGGAGACTGTGGGTGCATTC	
α6	F: GGTGACCGGGCATCCCAGTGA	197
	R: TGTTACAGCACCCCCAAATCCTGGC	
β1	F: ATCGAGAGAGTTTGGGGCTTC	80
	R: GCTGGGTTCATTGGAGCTGT	
β2	F: TAGTGGGCACGACGGTTAGA	111
	R: ATGACGATCCACCACAGCAG	
β3	F: TCTGAGCCCAGCAGCGTAAA	133
	R: TGTTCATCCCCACGCAGAC	
δ	F: AGGAACGCCATCGTCCTTTT	73
	R: CTTGACGACGGGAGATAGCC	
ε	F: ACTGCGCCCTGGCATTGGAG	70
	R: AGGCCCGAGGCTGTTGACAA	
γ1	F: CTCAGTTCTGCTGGGAGTCG	111
	R: CCCCAAGCACAGAGTTTTGC	
γ2	F: CTGAGCTGACGCTTTGATGG	120
	R: TGCCTCTAGTAGGTCCCGTC	
γ3	F: ATTACATCCAGATTCCACAAGATG	149
	R: CAC AGG TGT CCT CAA ATT CCT	
ρ1	F: CTTCTCACGGCTTCTTGGGA	98
	R: ACCCATCCCCACCACAAAAG	
ρ2	F: GAAGATTCGAAGACCTCCACCTCAGTC	120
	R: GTCTTTGTCCAGCTCTGTGATCTTCATTC	
ρ3	F: CAACTCAACAGGAGGGGAAA	101
	R: TCCACATCAGTCTCGCTGTC	
pi	F: TCGGTGGTGACCCAGTTCGGAT	115
	R: TCTGTCCAACGCTGCCGGAG	
σ	F: GCTGGAGGTGGAGAGCTATGGCT	115
	R: CCCCAGGTACGTGTACTGAGGGA	
GAT1	F: GCTTTCGGAAGTTGGGTGTG	135
	R: GTTGGACTGGAAAGGTGGTCT	
GAT3	F:TGTGCGGGCATCTTCATCTT	81
	R: GCCCCAAGCAGGATATGTGT	
BGT1	F: TTCTGGGAGAGACGGGTTTTG	158
	R: GCTGTGAAGTAAACAACCTTGC	
NKCC1	F: TCCTTCTCGGTGGACTGGTGGT	95
	R: AAGAGCTCGTCCTCATCGTCGC	
NKCC2 isoform A	F: GGTAACCTCTATCACTGGGT	154
	R: GTCATTGGTTGGATCCACCA	
KCC1	F: AACGAGGTCATTGTCACGCGCT	147
	R: ACGCACCAACAACACCCGTT	
KCC2	F: GTTCCATGTCCATCCAGGTGA	110
	R: ATTGCATTGCCCTGCACATAG	
KCC3	F: AGTGAAGATGCTCGCGCTTGGA	170
	R: AGCATGCCCCCATCATGCACAA	
KCC4	F: AACTGGCGTCCACGCTTCAAGT	122
	R: CCGGCAATGAGCATGGCGAAAA	
GABABR1	F: ATTTCCGATGTGACCCCGAC	102
	R: TTCGATTCACCTGGCAGTGG	
GABABR2	F: TCCGGAACGGGGAAAGAATG	136
	R: TCCGACCCCTGGAACCTTAT	
SDHA	F: GCCCATGCCAGGGAAGATTA	92
	R: TGTTCCCCAAACGGCTTCTT	
RPL13a	F: ATTGTGGCCAAGCAGGTACT	142
	R: CTCGGGAGGGGTTGGTATTC	
Ppia	F: CGCGTCTCCTTCGAGCTGTTTG	150
	R: TGTAAAGTCACCACCCTGGCACAT	

### Photothrombosis Model of Focal Ischemia

The University of Otago Animal Ethics Committee approved all animal experiments. Focal stroke was induced in the left hemisphere in either adult (2–3 month old, for protection studies) or aged (22 month old, for recovery studies) male C57BL/6J mice weighing 25–27 g (2–3 month old) or 32–40 g (22 month old) using the photothrombosis method (Clarkson et al., 2010, 2019). Briefly, mice were anesthetized and placed in a stereotactic apparatus, a midline incision was performed to expose the skull, which was subsequently cleared of connective tissue and dried. The cold light source (KL1500 LCD, Zeiss) was attached to a 40× objective to give a 2 mm diameter illumination and was positioned 1.5 mm lateral from Bregma. Rose Bengal solution (0.2 mL, Sigma; 10 g/L in normal saline, i.p.) was administered and the brain illuminated through the intact skull for 15 min after a 5 min lag period. The color temperature intensity used for all experiments to induce a stroke was 3300 K. Mice had unrestricted access to water and food and were housed under a 12-h light/dark cycle.

### Drug Administration

DS2 (0.01, 0.1, 1 or 4 mg/kg in DMSO) or vehicle (DMSO) was injected i.p., 1 h after stroke. A second dose was given 24 h later by an independent experimenter not involved in any other experiments in order to randomize the animals and minimize bias. For recovery studies, DS2 (10 mM) was loaded into ALZET 1002 mini-pumps and implanted subcutaneously from 3-days post-stroke. Mini-pumps were replaced after 2 week with new pumps to allow for continuous treatment for 28-days after stroke. The treatment groups were assigned randomly post-stroke.

### Behavioral Assessment

The baseline performance of animals was established 1 week prior to surgery, both, on the grid-walking and cylinder tasks. One week after stroke induction, animals were tested for motor function at the end of their dark cycle. For recovery studies, behavioral assessments were performed on weeks 1, 2, 4, and 6 post-stroke. Observers, blinded to the treatment group, scored the behaviors as previously described (Clarkson et al., 2010, 2019).

### Stroke Volume

Animals were anesthetized seven or 42 days post-stroke, and subsequently transcardially perfused with 4% paraformaldehyde, brains extracted and processed histologically. Cresyl violet staining was used to quantify stroke volumes as reported previously (Clarkson et al., 2010, 2011, 2019; Overman et al., 2012).

### Assessment of Circulating Pro-inflammatory Cytokine Levels

Three days post-stroke, blood samples were collected into EDTA treated tubes and centrifuged at 1000 × *g* for 10 min, 4°C. Plasma was collected and then stored at −80°C until required for analysis. A multiplex bead assay was performed using the Luminex 100^TM^ analyzer (Luminex Corporation, Austin, TX, United States). Cytokines IL-1β, TNF-α, IL-6, and IL-17 were analyzed with a BioPlex mouse cytokine assay kit (Bio-Rad Laboratories Inc., New Zealand), according to the manufacturer’s instructions. For each cytokine, a minimum of 100 events were collected. Cytokine concentrations were automatically calculated from median fluorescence intensities (MFI), based on standard curve data (detectable range of 2–32,000 pg/ml) using Bio-Plex Manager^TM^ software.

### Brain Accumulation of DS2 in Combination With Elacridar

Male black 6 mice (C57/BL/6Ntac, Taconic, Denmark) received i.v. administration of either elacridar (5 mg/kg, 1 mg/ml, in a dose volume of 5 ml/kg, dissolved in dimethyl acetamide/PEG400/30%/(2-hydroxypropyl)-beta-cyclodextrin (HPbCD) (10/40/50)) or vehicle. Thirty minutes later, DS2 [10 mg/kg, 1 mg/ml, in a dose volume of 10 ml/kg, dissolved in 5% dimethylsulfoxide (DMSO) + 30% HPbCD in demineralized water] was administered to mice orally. Animals were sacrificed and blood and brains were isolated at either 0.5 or 1 h post DS2 administration. DS2 concentrations in, plasma and brain tissue homogenates were determined using liquid chromatography-tandem mass spectrometry assay.

### Statistical Analysis

All data are expressed as mean ± SD. One-way analysis of variances (ANOVA) and Newman–Keuls’ multiple pair-wise comparisons for *post hoc* comparisons were applied for histological, biochemical DS2 brain/plasma levels and behavioral assessments post-stroke. For behavioral assessments in the recovery study, a two-way ANOVA following Tukey’s multiple pairwise comparisons performed. Five mice were used per treatment group for histological and seven mice per group for behavioral assessments. *P* < 0.05 was considered significant. Samples sizes were based on prior publications (Clarkson et al., 2010, 2013, 2019). To detect differences in infarct size in vehicle and DS2-treated mice, an *n* = 3 mice/group is sufficient for achieving a power of 80%, when using the following parameters: α = 0.05 (type 1 error rate); β = 0.2 (type 2 error rate); q1 = 0.5 (proportion of mice in vehicle group); q2 = 0.5 (proportion of mice in DS2 treated group); E = 2.94 (effect size, magnitude of difference); and S = 1.26 (Standard deviation).

## Results

### DS2 Is Neuroprotective and Improves Functional Recovery After Focal Ischemia

**FIGURE 1:**
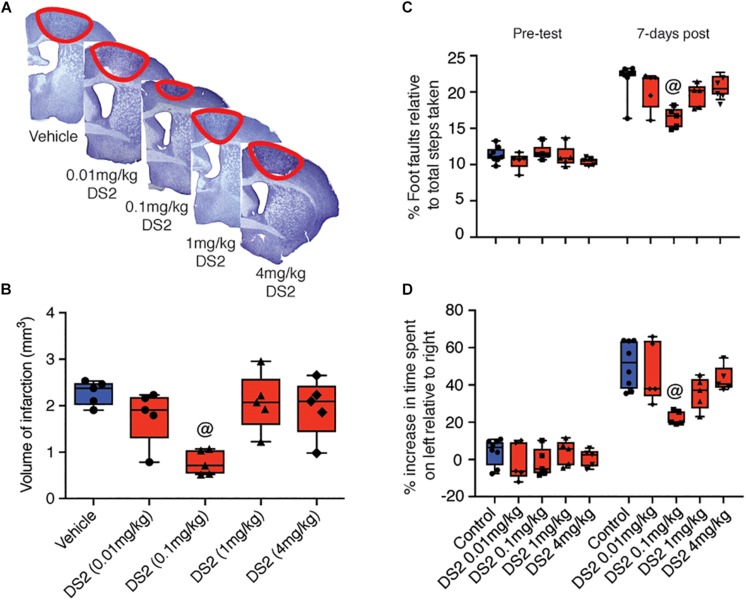
Treatment with DS2 reduces infarct volume and improves functional outcomes post-stroke. Mice were subjected to focal stroke and injected intraperitoneally with either DS2 (0.01, 0.1, 1 or 4 mg/kg) or vehicle 1 and 24 h after the surgery. The brains were collected 7 days later, stained with cresyl violet and the stroke volume was calculated. **(A)** Representative cresyl violet-stained sections of animals treated with vehicle or three different concentrations of DS2. **(B)** Mice injected with 0.1 mg/kg DS2 had significantly smaller strokes than untreated animals. Baseline motor function was established by grid-walking and cylinder test 1 week prior stroke surgery. Mice treated with either vehicle or DS2 (0.01 0.1, 1, and 4 mg/kg) at 1 and 24 h post-stroke induction were assessed in the **(C)** grid-walking (foot-faults) and **(D)** cylinder task (forelimb asymmetry). Data are expressed as mean ± SD for *n* = 5-7 per group. ^@^*P* < 0.01 compared with vehicle control.

It has previously been shown that reduction of extrasynaptic GABAergic signaling from 3 days after stroke improves functional recovery (Clarkson et al., 2010). In contrast, treatment with the flavonoid, 2’MeO6MF, which was shown to stimulate an increase in extrasynaptic GABA signaling, early after stroke affords significant neuroprotection (Clarkson et al., 2019). To further investigate the role of delta-containing GABA_A_ receptors as a potential target for stroke, we tested the delta-selective compound, DS2, for its ability to afford neuroprotection after stroke. The protective effects of DS2 were investigated in a focal photothrombosis model of stroke. The reperfusion component is minimal in this model and it is in general more difficult to protect against cell death. Mice were injected i.p. with DS2 (0.01-4 mg/kg) or vehicle 1 and 24 h post-stroke. Body temperature was stable (data not shown) and mice did not show signs of sedation. The stroke volume was assessed 7-days post-stroke and demonstrated that treatment with DS2 resulted in a decrease in infarct volume [*F*(4,20) = 6.912; *P* < 0.0012: vehicle, 2.28 ± 0.26 mm^3^ versus DS2 (0.01 mg/kg), 1.78 ± 0.58 mm^3^, *P* > 0.05; DS2 (0.1 mg/kg), 0.77 ± 0.27 mm^3^, *P* < 0.01, DS2 (1 mg/kg), 2.01 ± 0.62 mm^3^, *P* > 0.05, and DS2 (4 mg/kg) 1.96 ± 0.62 mm^3^, *P* > 0.05: *n* = 5/group; Figures 1A,B].

Next mice were tested for grid-walking (forelimb function) and cylinder (forelimb asymmetry) tasks. Consistent with what we have previously reported, mice had increased numbers of foot-faults on the grid-walking test (*n* = 5 per group; Figure 1C) and an increase in spontaneous forelimb asymmetry in the cylinder task (*n* = 5 per group; Figure 1D) was detected 7-days post-stroke (Clarkson et al., 2010, 2013, 2019). Treatment with DS2 decreased the number of foot-faults on the grid-walking task (*F*(4,20) = 4.06, *P* = 0.0144) and an improved forelimb asymmetry in the cylinder task (*F*(4,20) = 5.858, *P* = 0.0027). *Post hoc* analysis revealed that only treatment with 0.1 mg/kg DS2 significantly improved motor function on either the grid-walking (*P* = 0.0094) or cylinder (*P* = 0.0022) tasks, compared to vehicle-treated controls.

### DS2 Modulates Inflammatory Pathways

**FIGURE 2:**
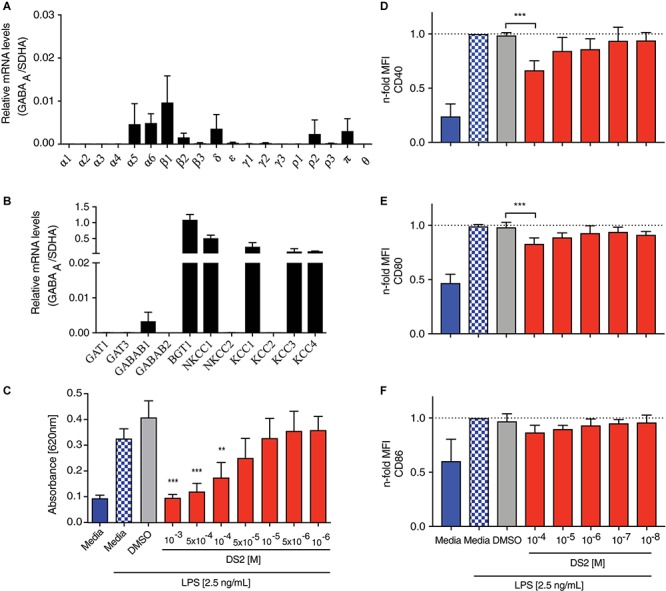
DS2 dampens activation of innate immune cells after an inflammatory stimulus. **(A,B)** Expression of GABA receptor subunits by RAWblue macrophages was analyzed by qPCR. Relative mRNA levels of **(A)** GABA receptor subunits and **(B)** transporters involved in GABA signaling on unstimulated RAWblue macrophages in relation to the reference gene succinate dehydrogenase complex subunit A (SDHA) are shown. **(C)** RAWblue macrophages (5 × 10^4^ cells/well) were either pre-treated with DS2 (10^–3^ to 10^–6^ M) or DMSO (2.5%) for 30 min or left un-treated before stimulation with LPS (2.5 ng/mL) for 6 h. Cell supernatants were collected and the extent of NF-κB activation was determined by quantifying the reporter protein SEAP with the Quantiblue^TM^ assay. **(D–F)** BMDCs were incubated with decreasing concentrations of DS2 (10^–4^ to 10^–8^ M) for 30 min prior to the addition of LPS (100 ng/mL) to the cultures. BMDCs were harvested 24 h later and expression of cell surface markers CD40, CD80 and CD86 on live, CD11c^+^ MHCII^+^ BMDCs was determined by flow cytometry. MFI shown as n-fold increase over LPS-treated cells. Results are the mean ± SD of three independent experiments with three replicates each. ^∗∗∗^*P* < 0.001 and ^∗∗^*P* < 0.01.

Inflammation is crucially involved in contributing to modulation and progression of tissue damage induced by the stroke. Inflammation contributes to the induction of apoptotic processes (Downes and Crack, 2010), and stimulates tonic GABA signaling (Wang et al., 2012), which hinders regaining function after stroke (Clarkson et al., 2010). To confirm that innate immune cells express GABA receptor subunits, necessary for responding directly to DS2, qPCR was performed on unstimulated RAWblue macrophages for the following GABA_A_ receptor subunits (α1, α2, α3, α4, α5, α6, β1, β2, β3, δ, ε, γ1, γ2, γ3, ρ1, ρ2, ρ3, π, θ), GABAB receptor subunits (GABAB1, GABAB2) and GABA transporters (GAT1, GAT3, BGT1, NKCC1, NKCC2, KCC1, KCC2, KCC3, KCC4) involved in GABA signaling. The relative mRNA levels were investigated by qPCR using the specific primers listed in Table 1. We detected several GABA_A_ receptor subunits, with β_1_, α_5_, α_6_, and δ showing the highest relative expression (Figure 2A). mRNA levels for the subunits β_2_, π, and ρ_2_ were lower in comparison but higher than the levels of the low-expressing subunits β_3_, ε, γ_1__–__2_, and ρ_3_. In addition, high levels of transcripts for the betaine/GABA receptor (BGT1), the K^+^-Cl^–^ co-transporters KCC3 and KCC4 and the Na^+^ K^+^ Cl^–^ co-transporter NKCC1 were found in RAWblue macrophages (Figure 2B). The mRNA levels of GABA receptor subunits and transporters were not significantly changed upon stimulation with LPS for either 6 or 24 h (data not shown).

Next, we assessed the efficacy of DS2 to decrease NF-kB activity, a common measure of inflammation. RAWblue macrophages, stimulated with LPS (2.5 ng/mL), showed a marked increase in NF-kB activity (*P* < 0.001; Figure 2C), which did not change upon addition of the vehicle (2.5% DMSO). LPS-stimulated macrophages, pre-incubated with DS2 (10^–3^ to 10^–4^ M) showed a significant decrease in NF-kB activity (*P* < 0.001; Figure 2C) with levels being similar to unstimulated macrophages.

To confirm the anti-inflammatory effect of DS2, observed in the immortalized RAWblue macrophage cell line, we next tested DS2 using LPS-stimulated primary innate immune cells. For this experiment we generated BMDCs from bone marrow precursor cells by adding GM-CSF to the culture medium. Resulting CD11c^+^ MHCII^+^ BMDCs have previously been shown to closely resemble migratory DCs found *in vivo* (Helft et al., 2015), which makes them an interesting population to study in the stroke context. BMDCs were pre-incubated with varying concentrations of DS2 before LPS was added for 24 h. BMDCs were harvested and the expression of CD40, CD80, and CD86 determined by flow cytometry. BMDCs pre-incubated with the highest dose of DS2 (10^–4^ M) demonstrated a significant decrease in CD40, CD80 (*P* < 0.01) but not CD86 expression when stimulated with LPS (Figures 2D–F). Treatment with DS2 did not affect the viability of RAWblue macrophages and BMDCs (data not shown). This data further confirms that DS2 is having a positive effect to dampen the immune response.

### DS2 Dampens Stroke-Induced Cytokine Production *in vivo*

**FIGURE 3:**
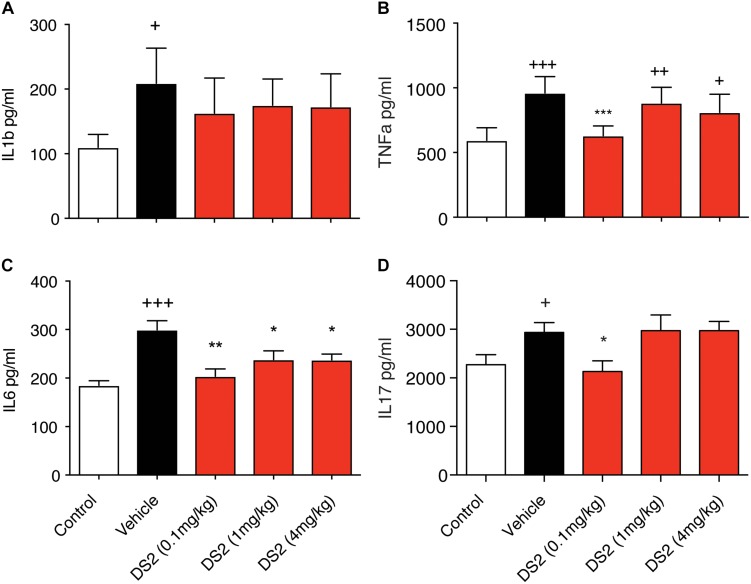
DS2 decreases pro-inflammatory cytokine production post-stroke. Mice were injected i.p. with vehicle or DS2 (0.1, 1 or 4 mg/kg) 1 and 24 h after induction of stroke and peripheral blood collected from the tail vein of mice 3-days post-stroke. Sera were analyzed for **(A)** IL-β, **(B)** TNF-α, **(C)** IL-6, and **(D)** IL-17 concentrations using a BioPlex kit. Data are expressed as mean ± SD for *n* = 5 per group. ^∗∗∗^*P* < 0.001, ^∗∗^*P* < 0.01, ^∗^*P* < 0.05 compared to vehicle. ^+++^*P* < 0.001, ^++^*P* < 0.01, ^+^*P* < 0.05 compared to sham control.

Next, we investigated the effects of DS2 on inflammation post-stroke *in vivo*. As reported previously, we show that cerebral ischemia significantly increases peripheral cytokine levels of IL-1β (sham control versus stroke + vehicle: 109.91 ± 20.08 pg/mL versus 209.24 ± 54.05 pg/mL; *P* < 0.01, Figure 3A), TNF-α (sham control versus stroke + vehicle: 594.02 ± 97.44 pg/mL versus 960.98 ± 125.63 pg/mL; *P* < 0.001, Figure 3B), IL-6 (sham control: 185.33 ± 20.17 pg/mL versus stroke + vehicle: 299.50 ± 41.52 pg/mL; *P* < 0.001, Figure 3C), and IL-17 (sham control: 2145.31 ± 124.27 pg/mL, versus stroke + vehicle: 2934.50 ± 204.76 pg/mL; *P* < 0.05, Figure 3D).

Treatment with DS2 significantly decreased circulating IL-6 (0.1 mg/kg, *P* < 0.01; 1 and 4 mg/kg, *P* < 0.05), IL-17 and TNF-α levels (*P* < 0.05 and *P* < 0.001, respectively following treatment) at 3-days post-stroke. Treatment however, with DS2 did not have any effect on IL-1β levels with levels remaining similar to stroke + vehicle controls. These data suggest that DS2 can dampen an NF-kB-mediated pro-inflammatory response that is most likely occurring via δ-containing GABA_A_ receptors located on immune cells.

### DS2 Shows Limited Brain Exposure Following Systemic Administration

To correlate our *in vitro* data with our *in vivo* data, we assessed the ability of DS2 to cross the mouse BBB. We found that injection of a single dose of DS2 (i.p.,10 mg/kg), resulted in robust plasma, but only marginal brain exposure at either 0.5 or 1 h following dosing (Table 2). The free fraction of DS2 in brain tissue is very low (1.2% unbound, Saniona, unpublished data), corresponding to 54 and 64 nM free at 0.5 and 1 h following dosing respectively, which does not cover the ED50 for potentiating the effect of GABA at the α4β3δ receptors in L(tk-) cells (Wafford et al., 2009). As DS2 was speculated to act as a P-glycoprotein substrate, which could block the entry of DS2 into the brain, we next assessed if co-administration of DS2 with elacridar, an inhibitor of both p-glycoprotein and breast cancer resistance protein (BCRP) (Cutler et al., 2006), would increase brain DS2 levels. Assessment of both brain and plasma DS2 levels in the presence of elacridar revealed similar levels at both 0.5 and 1 h following DS2 dosing as levels reported without elacridar, indicating that brain penetrance by DS2 is not restricted by active efflux by p-glycoprotein or BCRP (Table 2). These results suggest that DS2, in the dose tested, does not lead to sufficient free brain concentrations needed to activate the α4β3δ receptors indicating that the protective effects of DS2 most likely occur via modulation of the peripheral immune response or some as yet unknown mechanism. We also acknowledge that the BBB is leaky after stroke and more so in animals relative to humans, which could lead to mildly higher brain concentrations. However, in a recent study using a GABA modulator, we did not observe this (Clarkson et al., 2019).

**TABLE 2:** Brain and plasma concentrations following DS2 administration and co-treatment with or without elacridar.

		**Plasma (P)**		**Brain (B)**		**P ratio**	**B ratio**	**B/P ration**	
		**Without ng/ml (μM)**	**With ng/ml (μM)**	**Without ng/ml (μM)**	**With ng/ml (μM)**			**Without**	**With**
DS2	0.5 h	12,205	12,262	1587	1696	1.0	1.1	0.13	0.14
		(34.5)	(34.7)	(4.5)	(4.8)				
	1 h	15,231	13,928	1874	1957	0.9	1.0	0.12	0.13
		(43.2)	(39.5)	(5.3)	(5.5)				

*Data is expressed as both ng/ml and (μM) concentrations.*

### DS2 Improves Functional Recovery When Administered at a Delay

**FIGURE 4:**
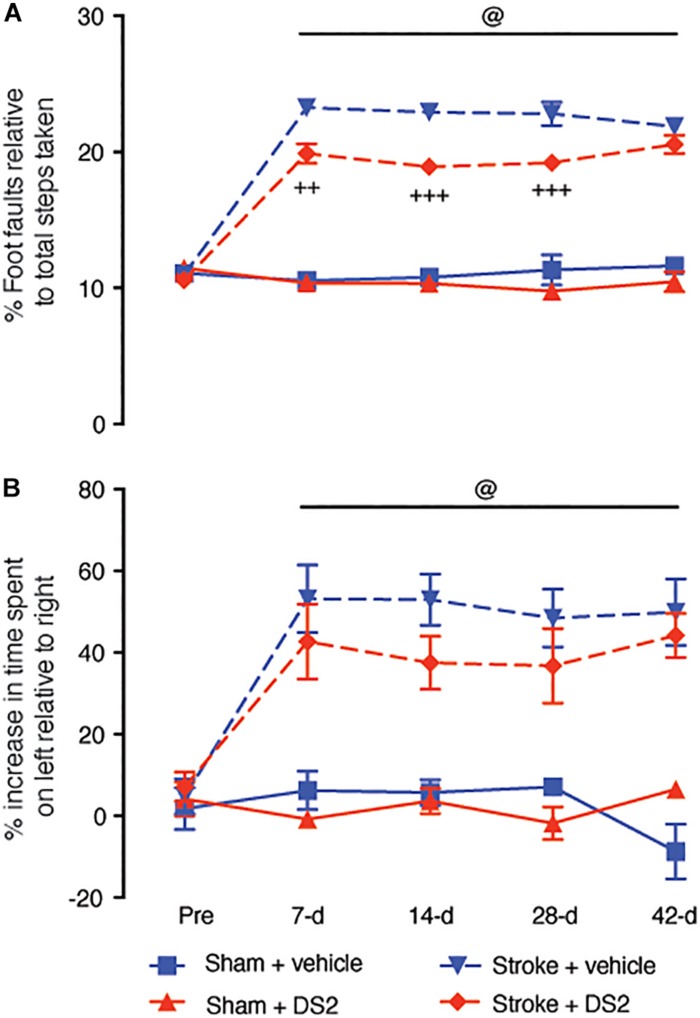
Delayed administration of DS2 from day-3 post-stroke improves functional outcomes when continuously administered. Mice were subjected to focal stroke or sham surgery and DS2 (10 mM, red) or vehicle (blue) were loaded into ALZET 1002 mini-pumps and implanted subcutaneously from 3-days post-stroke. Mini-pumps were replaced after 2 week with new pumps to allow for continuous treatment for 28-days after stroke. Mice were assessed for functional deficits using the **(A)** grid-walking (foot-faults) and **(B)** cylinder task (forelimb asymmetry). Data are expressed as mean ± SD for *n* = 7 per group. ^+++^*P* < 0.001, ^++^*P* < 0.01 compared to stroke vehicle control. ^@^*P* < 0.001 compared to sham controls.

Finally, as DS2 appears to dampen peripheral immune activation and only showed limited brain exposure, we assessed the role of DS2 to promote functional recovery after focal stroke to the motor cortex when administered at a delay of 3 days. DS2 was administered continuously from 3 to 28-days post-stroke, a critical period for neuroplasticity in rodents post-stroke (Corbett et al., 2017). This is also a period where we have previously reported that starting treatment with GABA modulators can aid in improving functional recovery without affecting stroke volume (Clarkson et al., 2010; Lie et al., 2017). Throughout the 42 day observation period, we detected marked forelimb motor deficits in the grid-walking and cylinder tasks in mice treated with vehicle only. Vehicle-treated mice showed limited spontaneous recovery (Figures 4A,B).

Chronic delayed treatment with DS2 improved motor performance of the affected forelimb on days 7, 14, and 28 post-stroke on the grid-walking task (Figure 4A). However, upon discontinuation of treatment, the DS2 treated animals showed a decrease in the recovery, such that no difference was observed in grid-walking performance at 42-days post-stroke compared with the vehicle-treated controls recovering from the stroke. Analysis of cylinder task performance showed no significant improvement in the presence of DS2 compared to vehicle treated controls (Figure 4B). However, it should be noted that some animals did show an improvement.

Delayed DS2 treatment did not induce any obvious signs of seizures or seizure-like behaviors in any of the mice throughout the 42 days. In addition, delayed treatment had no effect on infarct volumes (data not shown).

## Discussion

It is currently not fully understood how GABAergic compounds promote neuroprotection after stroke. GABA_A_ receptor subtype-selective agonists, such as DS2 provide an opportunity to investigate the mechanism of action more thoroughly. Receptor subtype-selective compounds are more likely to have an effect and have therefore been investigated clinically for treating diseases, such as schizophrenia, Downs syndrome and autism (Rudolph and Knoflach, 2011; Rudolph and Mohler, 2014). Furthermore, using subtype selective GABAergic compounds will allow us to increase our understanding of GABA signaling post-stroke. In addition to the positive allosteric GABA_A_ modulator described here, we have previously reported that negative modulation of GABA_A_ receptors can improve stroke recovery when administered three days post-stroke (Clarkson et al., 2010). Within this timeframe, the infarct is fully developed and pharmaceutical treatments aimed to improve cortical excitability do not necessarily change the infarct size (Clarkson et al., 2010, 2011). These findings have led to pharmaceuticals companies developing compounds that aim to normalize extrasynaptic GABA_A_ inhibition, and assess these for stroke in human Phase II clinical trials (ClinicalTrials.gov ID: Hoffman La-Roche – NCT02928393; Servier RESTORE BRAIN Study – NCT02877615).

Activation of GABA_A_ receptors can mediate both synaptic and extrasynaptic inhibitory currents depending on the receptor composition (Sieghart, 1995). α5- or δ-subunits are crucial components of extrasynaptic GABA_A_ receptors in the hippocampus, cortex and thalamus (Glykys and Mody, 2007; Olsen and Sieghart, 2009). Antagonism of α5- or δ-subunits facilitates pyramidal neuron signaling to afferent inputs (Drasbek and Jensen, 2006; Glykys and Mody, 2007), and enhances neuronal network excitability (Walker and Semyanov, 2008). Since extrasynaptic GABA_A_ receptors play an important role in shaping neuronal excitability, modulation of α5- or δ-subunits early could offer a viable treatment for reducing the glutamate-mediated hyperexcitability, which we have recently reported following treatment with the flavonoid, 2’MeO6MF (Clarkson et al., 2019). As flavonoids can have multiple modes of action it is good to assess the mechanistic function of δ-subunit-mediated protection using other δ-subunits modulators, such as DS2 (Wafford et al., 2009) or AA29504 (Vardya et al., 2012).

Prior studies have shown that DS2 binds to and modulates δ-subunit containing extrasynaptic GABA_A_ receptors in neuronal cell culture systems (Wafford et al., 2009; Jensen et al., 2013; Lee et al., 2016). DS2 enhances the maximum GABA response at α4/6βxδ GABA_A_ receptors while only a minimal response is observed at α4/6βxγ2 GABA_A_ receptors, clearly indicating a δ-subunit preference of DS2. While the potency of DS2 is influenced by which α-subunit is present in the receptor complex (α4/6βxδ > α1βxδ >> γ2-GABAA receptors), the type of β-subunit does not affect the potency as much (Wafford et al., 2009; Ahring et al., 2016). Our findings suggest that the effects of DS2 on macrophages are most likely mediated via binding to α6β1/2δ GABA_A_ receptors. Previously, α6βδ-subunit containing GABA_A_ receptors have been identified in cerebellar granule cells (Nusser et al., 1998) whereas α4βδ receptors were found in hippocampal interneurons (Barnard et al., 1998).

Our study is one of the first to assess the biological efficacy of DS2 *in vivo*, and in doing so confirm the observation by Jensen et al. (2013) that DS2 has a limited ability to cross the BBB. In the studies by Jensen and colleagues, treatment with DS2, even at doses 10-fold higher than what we used in the current study, DS2 failed to show any biological response when assessed for gross behavior, psychosis and pain. While the brain concentration we report of 1587 ng/ml (4.5 μM) following a single 10 mg/kg i.p. dose appears modest, the free fraction is only 1.2% of this and therefore, equates to a 45 nM dose equivalent to an ED1-ED5 for potentiating the effect of GABA at the α4β3δ receptors. In our studies, we observed a significant reduction of stroke infarct size and an improvement of functional outcomes after administration of two doses of 0.1 mg/kg DS2. According to our pharmacokinetic studies it can be expected that this dose would result in a total and free plasma concentration of 345 and 4.1 nM, respectively. The total and free DS2 brain concentration are estimated to be 45 and 0.54 nM after a dose of 0.1 mg/kg DS2. Such low brain concentrations are unlikely to elicit a biological effect. It still remains to be determined whether these low concentrations of DS2 can exert a local effect on extrasynaptic GABA_A_ receptors. The lack of efficacy of DS2 at higher doses could be due to a couple of reasons: (1) potentially desensitization of the delta subunit at these slightly higher doses, which means DS2 may switch from having an agonist effect to having an antagonist effect, but most likely (2) the biphasic dose response is due to hormesis (Calabrese et al., 2017). The term hormesis, which means “to excite,” was first described when researchers found some fungi from Red Cedar trees displayed a stimulatory effect at low-doses whilst having an inhibitory effect at high-dose for cell metabolism and survival (Calabrese et al., 2017). Such hormetic dose-responses have been reported previous for stroke, but mainly in a neuroprotective/preconditioning setting (Bradley et al., 2014). In addition, similar hormetic responses have also been extensively reported for cancer and inflammatory conditions (Calabrese, 2005). The mechanisms that underpin hormetic responses is poorly understood, but it is known that changes in intracellular signaling pathways are also altered and it could be an intrinsic cellular response that ensures neuronal activity does not get too out of balance. This is clearly an area that requires much needed work, especially when it comes to drug receptor interactions and pharmacokinetic responses.

In this study, we demonstrate that DS2 can act by dampening immune cell activation and inflammation, offering this as a novel mechanism of action for DS2. Innate and adaptive immune cells produce functional GABA_A_ receptors (Fuks et al., 2012) where binding of GABA has been demonstrated to reduce inflammation (Reyes-Garcia et al., 2007). Mechanistically this has been linked to a GABA-mediated decrease in IL-1β, IL-6, and IL-12 production following LPS-stimulation of innate immune cells, including peritoneal macrophages (Reyes-Garcia et al., 2007; Bhat et al., 2010). Here we add to these observations by showing that the δ-subunit preferring GABA_A_ agonist, DS2, reduced LPS-induced NF-kB activation in a murine macrophage cell line and decreased expression of co-stimulatory molecules in primary BMDCs. The influx of DCs into the brain increases dramatically post-stroke and is associated with elevated expression of co-stimulatory molecules MHCII, CD40, and CD80 (Gelderblom et al., 2009; Posel et al., 2014). The induction of these co-stimulatory molecules occurs following exposure to DAMPs that are derived from dying and necrotic cells in the post-ischemic hemisphere (Offner et al., 2006; Gelderblom et al., 2009; Felger et al., 2010). Mature DCs are able to present antigens on MHC molecules to T-cells (signal 1) and provide co-stimulation through CD40, CD80, and CD86 expression (signal 2) and cytokine secretion (signal 3). The initiation of an adaptive immune response has been linked to development of autoimmunity and worse outcomes in stroke patients (Becker et al., 2011). Reduced expression of the co-stimulatory markers seen after treatment of BMDCs with DS2 may therefore translate into decreased ability of DCs to stimulate autoimmune responses in the post-ischemic brain. Since administration of the δ-subunit-selective compound, DS2, led to a decreased inflammatory response in murine macrophages and BMDCs, it is tempting to speculate that the δ-subunit is a crucial component for elucidating anti-inflammatory responses through GABA_A_ receptors. This is supported by studies showing good expression levels of the δ-subunit in a range of murine and human innate immune cells, such as peritoneal macrophages (Reyes-Garcia et al., 2007), human peripheral blood mononuclear cells (Alam et al., 2006), monocytes and immortalized monocytic cell lines (Wheeler et al., 2011).

Interestingly, expression of GABA_A_ receptors and in particular the δ-subunit has also been found in resting, activated and encephalitogenic T-cells (Tian et al., 2004; Bjurstom et al., 2008). Even though it remains to be determined if DS2 has a direct effect on T-cells it is likely that DS2 modulates T-cells indirectly. Bhat et al. (2010) have previously demonstrated a direct effect of GABAergic agents on innate immune cells, which did not affect cytokine production or proliferation of T-cells directly. However, cytokines secreted by innate immune cells altered the function of T-cell, which was associated with delayed onset and reduced severity in an animal model of multiple sclerosis. This links a reduced activity of innate immune cells with downstream T-cell responses, known to have a major impact on neurological diseases (Brait et al., 2012; Anderson et al., 2014).

The production of pro-inflammatory cytokines and expression of activation markers on innate immune cells has been previously observed to impact the priming of naïve T-cells and drives the polarization of T-cells toward a distinct effector function (Martin-Fontecha et al., 2003; Joffre et al., 2009). In particular, polarization of γδ T-cells and αβ T-cells toward IL-17-producing subsets has been shown to negatively impact stroke recovery (Shichita et al., 2009). T-cells, producing IL-17 have been found in increased numbers in the blood of patients early after stroke onset and has also been observed in post-mortem brain tissue of stroke patients (Kostulas et al., 1999; Gelderblom et al., 2012). IL-17 also contributes to disruption of BBB tight junctions and neutrophil recruitment into the brain, resulting in further tissue damage (Kebir et al., 2007; Wang et al., 2009; Gelderblom et al., 2012). We report here that systemic administration of DS2 decreases blood cytokine levels of, IL-6, TNF-α, and IL-17, three days post-stroke, which suggests that DS2-mediated suppression of innate immune cell activation could be associated with reduced numbers of Il-17 producing cells infiltrating the brain. It should be noted however, that not all cytokines are suppressed, as IL-1β is still elevated indicating that part of the innate immune system is most likely still active, the extent of which requires further investigation.

In addition to influencing immune cell phenotypes, inflammatory cytokines, such as TNF-α are able control the extent of immune cell entry into the CNS at the choroid plexus epithelium (Kunis et al., 2013). Here, TNF-α and interferon-γ synergistically promote the expression of trafficking molecules that promote the entry of leukocytes into the CNS under conditions of inflammation. Based on our data, it is possible to assume that the dampened systemic TNF-α levels following treatment with DS2, may inhibit the trafficking of immune cells through the choroid plexus and into the CNS. The reduction in systemic cytokine levels may also be interesting in the context of the recent discovery that innate immune cells originate from the skull bone marrow and migrate to the brain after ischemic injury (Herisson et al., 2018). The skull bone marrow can be reached by the systemic blood supply, containing drugs, such as DS2 and thus potentially impact the trafficking of leukocytes via modulation of cytokine and chemokine expression.

We show here that administration of DS2 attenuates peripheral cytokine levels, associated with inflammation. Recent work demonstrated that pro-inflammatory cytokines, such IL-1β affect the surface expression of GABA_A_ receptor subunits, such as α_5_, which impaired memory (Wang et al., 2012). We observed using DS2 that this also applies to δ-containing GABA_A_ receptors. Increased cytokine levels could drive a delayed increase in extrasynaptic GABA signaling post-stroke (Clarkson et al., 2010). The relationship between blood cytokine levels and changes in extrasynaptic GABA currents has been reported, however, the mechanisms linking the two remain poorly understood. This could be happening in part via Phosphatidylinositol 3-Kinase/Akt signaling (Serantes et al., 2006). A change in cytokine production would most likely be mediated by the compound acting on immune cells directly and reducing the secretion of cytokines and not via alterations in GABA signaling. In support of this, we report that, DS2 show only limited brain exposure, and most likely dampens NF-kB activity in immune leading to reduced release of cytokines. This in turn could prevent the infiltration of immune cell and release of cytokines in the brain to alter GABA inhibitory currents. Increasing extrasynaptic GABA signaling does not trigger inflammatory processes, however, increased blood cytokine levels can elevate extrasynaptic GABA signaling. For this reason, compounds reducing cytokine production can be used within a day of stroke onset to reduce tissue damage. In addition, as the immune cell/inflammatory cascade is elevated across a protracted time window, starting treatment from day 3 post-stroke also modestly improve functional recovery. Interestingly, DS2 failed to dampen all pro-inflammatory cytokines, so the elevated IL-1β levels still present could possibly have an effect tonic inhibitory currents and still impair some recovery from happening.

In summary, we show here that increasing GABA_A_ signaling early provides protection, improves motor function and reduces peripheral cytokine levels associated with inflammation. In addition, delaying treatment until 3-days post-stroke resulted in a modest effect to improve functional recovery. In summary, our results identify GABA_A_ receptors containing the δ subunit as a novel target for stroke therapy and suggest peripheral inflammation as an important factor in driving cell death and impairing recovery post-stroke. However, it should be noted that dosing at these sites does appear to illicit a hormetic dose-response and the mechanisms that underpin this are yet to be ascertained.

## Data Availability Statement

The datasets generated for this study are available on request to the corresponding author.

## Author Contributions

SN, LB-B, EG, TJ, and AC acquired and analyzed the data, and critically revised the manuscript. SN, PA, KS-N, SY, and AC designed the experiments, analyzed the data, and prepared and edited the manuscript. All authors reviewed and approved the final version of the manuscript and have also agreed to be accountable for the content of the work.

## Conflict of Interest

TJ and KS-N are employed by Saniona A/S. The remaining authors declare that the research was conducted in the absence of any commercial or financial relationships that could be construed as a potential conflict of interest.
